# Alveolar ridge preservation and primary stability as influencing factors on the transfer accuracy of static guided implant placement: a prospective clinical trial

**DOI:** 10.1186/s12903-020-01155-x

**Published:** 2020-06-29

**Authors:** Sigmar Schnutenhaus, Liesa Brunken, Cornelia Edelmann, Jens Dreyhaupt, Heike Rudolph, Ralph G. Luthardt

**Affiliations:** 1 Zentrum für Zahnmedizin Dr. Schnutenhaus MVZ GmbH [Center for Dentistry Dr. Schnutenhaus Community Health Center (CHC) GmbH], Breiter Wasmen 10, 78247 Hilzingen, Germany; 2 Department for Dentistry, Clinic for Prosthodontics, Universität Ulm, Department für Zahnheilkunde, Klinik für Zahnärztliche Prothetik [Ulm University, Albert-Einstein-Allee 11, 89081 Ulm, Germany; 3 Universität Ulm, Institut für Epidemiologie und Medizinische Biometrie [Ulm University, Institute of Epidemiology and Medical Biometry], Schwabstr. 13, 89075 Ulm, Germany

**Keywords:** Dental implant, Surgical template, CBCT, Computer-guided surgery, Accuracy, Alveolar ridge preservation

## Abstract

**Background:**

The aim of this prospective clinical study was to investigate differences between virtually planned and clinically achieved implant positions in completely template-guided implant placements as a function of the tooth area, the use of alveolar ridge preservation, the implant length and diameter, and the primary implant stability.

**Methods:**

The accuracy of 48 implants was analyzed. The implants were placed in a completely template-guided manner. The data of the planned implant positions were superimposed on the actual clinical implant positions, followed by measurements of the 3D deviations in terms of the coronal (dc) and apical distance (da), height (h), angulation (ang), and statistical analysis.

**Results:**

The mean dc was 0.7 mm (SD: 0.3), the mean da was 1.4 mm (SD: 0.6), the mean h was 0.3 mm (SD: 0.3), and the mean ang was 4.1° (SD: 2.1). The tooth area and the use of alveolar ridge preservation had no significant effect on the results in terms of the implant positions. The implant length had a significant influence on da (*p* = 0.02). The implant diameter had a significant influence on ang (*p* = 0.04), and the primary stability had a significant influence on h (*p* = 0.02).

**Conclusion:**

Template-guided implant placement offers a high degree of accuracy independent of the tooth area, the use of measures for alveolar ridge preservation or the implant configuration.

A clinical benefit is therefore present, especially from a prosthetic point of view.

**Trial registration:**

German Clinical Trial Register and the International Clinical Trials Registry Platform of the WHO: DRKS00005978; date of registration: 11/09/2015.

## Background

A 3D-based preoperative diagnosis allows a detailed assessment of existing bone in all spatial dimensions. This facilitates planning of the optimal position and number of implants while taking into consideration the adjacent structures, e.g., the inferior alveolar nerve, maxillary sinus or adjacent teeth [1]. In addition, the prosthetic goal is defined using this implant planning with regard to function and esthetics. A reliable method is required for the precise transfer of the virtual design into the operative findings. Computer-assisted, static guided implant placement is an established procedure with a high predictability for the prosthetic final outcome. Moreover, the guided approach often allows a less invasive surgical approach. For this reason, the procedure must be clinically evaluated to guarantee satisfactory precision and protection of vulnerable structures in practice [2]. Inaccuracies can occur during all diagnostic and therapeutic steps. It must be possible to perform the individual steps as precisely as possible so that the total of all errors ultimately results in a clinically tolerable deviation of the implant position [3].

The transfer accuracy and influencing factors are already objects of a multitude of published studies and review papers. A comparison of guided implant placement with conventional free-hand implant placement demonstrated significantly more precise outcomes in favor of the guided approach [4–7]. Tahmaseb et al. examined 20 clinical studies with regard to the accuracy of static guided implant placement. The average deviation of all studies totaled 1.2 mm at the implant shoulder and 1.4 mm at the implant apex. The average angular deviation was 3.5° [8].

For successful, long-term stability of the implant placement, adequate bone availability is a key factor [9]. There are changes in the resorption characteristics of the alveolar process after tooth extractions [10]. A significant loss of bone volume specifically occurs in the buccal part of the alveolus. Within the first 6 months, there is a mean horizontal degeneration of the alveolar process of 3.8 mm and a mean vertical degeneration of 1.2 mm [11]. This bone loss presents a problem for the subsequent implant treatment and can result in severe restrictions in esthetics, phonetics, and function [12]. The technique of alveolar ridge preservation (ARP) with a bone replacement material mitigates the physiological dimensional change in the bone, which is a typical outcome of tooth extraction [13]. Study outcomes show a significant benefit of hard- and soft-tissue management compared to spontaneous healing [12, 14]. The indirect influence of measures taken in the area of the hard and soft tissue for alveolar ridge preservation on the bone structure before template-guided implantation has not yet been described in the literature. There are no clinical data on accuracy according to ARP and the dependence of such accuracy on bone structure, measured with primary stability.

Therefore, the objective of this prospective clinical study was to evaluate the transfer accuracy of fully guided implant placement using stereolithographic insertion guides. The outcomes were investigated with regard to the influence of the factors “tooth region, application of measures of the hard tissue management in the form of ARP, implant diameter, implant length, and primary stability (ISQ [Implant Stability Quotient])” on the accuracy. The primary stability of an implant depends on, among other things, the bone structure, the macro design of the implant and the surgical technique. The primary stability values expressed as measured by the ISQ and the screwing torque are strictly correlated with each other [15].

The proposed hypothesis was that neither the use of a collagen material for ARP nor the tooth region nor the implant length or diameter have an influence on the accuracy of implant placement. In particular, the study aimed to clarify whether there is a connection between the screwing torque, indirectly represented by the primary stability (ISQ). The null hypothesis was that the screwing resistance would not affect the accuracy of the implantation.

## Methods

### Patient selection

The data were collected as part of a prospective, randomized clinical study with a total of 60 patients. All patient data were collected after approval of the Ethics Committee of Ulm University (Application No. 337/12 and 41/14) in the period of 03/13/2014 to 09/05/2017 in the practice of PD Dr. Schnutenhaus in Hilzingen (Cooperation partner Ulm University, Department for Dentistry, Clinic for Prosthodontics). The subjects were allocated into two groups (A and B) based on a randomization list. For the entire study, a randomization list was created, in which 60 patients (Institute of Epidemiology and Medical Biometry, Ulm University, Ulm, Germany) were assigned to groups in 6 strata. The data were stratified by sex (male/female) and tooth region (anterior tooth, premolar, and molar). Patient information (gender, region) was submitted to the principal investigator (RGL) or an authorized individual (HR), who had blinded access to the randomization list. The random assignment information was sent to the treatment center by fax according to the randomization list. With the subjects of test group A, an ARP procedure was performed after tooth extraction; the extraction alveoli of control group B healed without further measures. The regions were divided into anterior teeth, premolar, and molar areas.

The following inclusion criteria were applied:

- Medically indicated extraction of a tooth in the upper jaw
- Treatment of the missing tooth by installing an implant
- At least one tooth or existing implant in the immediate vicinity of the tooth to be extracted

The exclusion criteria were as follows:

- Age under 18 years or lack of legal competence
- Impossibility of using an implant guide (restricted mouth opening)
- Necessary additional augmentation requirement
- Heavy smoking (> 10 cigarettes/day)
- Administration of bisphosphonates
- Pregnancy
- Alcohol and/or drug abuse
- Presence of infectious disease, e.g., hepatitis, HIV, or AIDS
- Poorly managed diabetes mellitus.

### Surgical protocol after tooth extraction

**Fig. 1:**
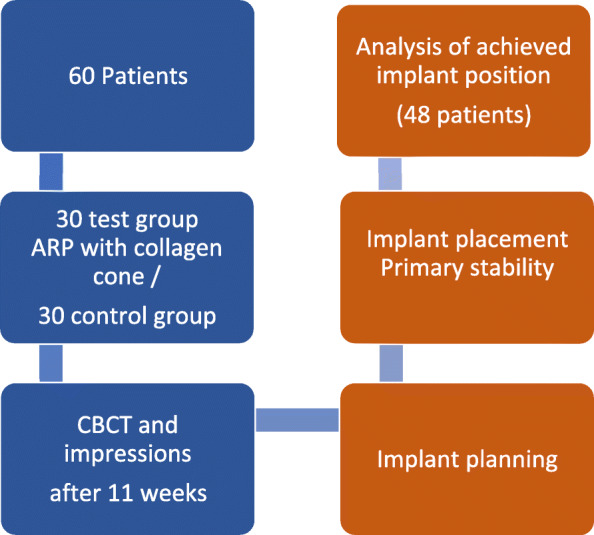
Workflow of alveolar ridge preservation and subsequent implant placement

With each of the 60 patients, tooth extraction in the upper jaw took place with subsequent treatment of the resulting gap with implanted prosthetics (Fig. 1). As part of a previous clinical study (“Resorba,” Ethics App. NO. 337/12), an impression of the extraction alveoli was made following the extraction, and this impression was scanned (3Shape Scanner D 700, 3Shape A/S, Copenhagen, Denmark). The subjects of the test group received an ARP in the form of a collagen cone in conjunction with a collagen membrane (Parasorb Sombrero, Resorba medical GmbH, Nuremberg, Germany). In the control group, healing without additional measures was awaited.

### Implant planning

**Fig. 2:**
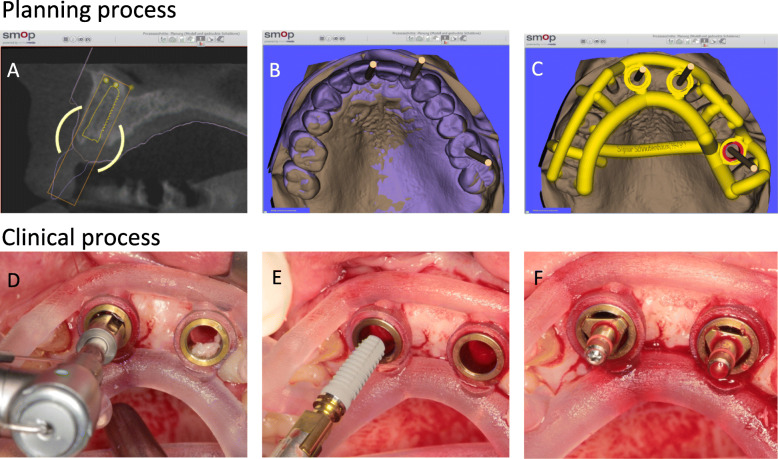
Clinical procedure of guided surgery: **a** and **b** Optimal implant position after prosthetic alignment and bone supply. **c** Template design. **d** Drilling through the template. **e** Insertion of the implant. **f** Final position of the implant

Cone beam computed tomography (CBCT) (Gendex CB500, Gendex Dental Systems, Des Plaines, USA) was performed with a resolution of 0.2 voxels 11 weeks after tooth extraction according to the protocol of early implantation (12–16 weeks after extraction). The 3D implant planning took place using the implant treatment planning software SMOP (SwissMeda OperationsPlanung, Swissmeda, Zurich). A diagnostic plaster model as well as prosthetic wax-up for each patient were optically scanned (3Shape Scanner D 700, 3Shape A/S, Copenhagen, Denmark) for implant planning. The facilitated superimposition of the CBCT data with the STL datasets of the patient model cast on the basis of a corresponding program function of the planning software. The dentist (SiS) manually made the fine-tuning adjustments of the superimposition. The optimal implant position was determined based on the information obtained on the bone availability, the soft tissue situation, and the planned prosthetic treatment. Implant planning was saved in the SMOP software. The template was then virtually designed by experts in Swissmeda during the CAD/CAM process using the same software (SMOP). It was stereolithographically produced in a 3D printer (Objet Eden 260 V, Material: MED610, Stratasys Ltd., Minneapolis, MN USA). There was only one center for template production and one printing process applied to reduce process-related inaccuracies. This process has been evaluated and published in several studies [16]. The same dentist (SiS) performed all design steps and the subsequent implant placement (Fig. 2).

### Surgical protocol during implant placement

Three months after the tooth extraction, the implant was inserted during a fully guided procedure using a drilling guide with dental support. All surgical procedures were performed using local anesthesia. The implant bed was prepared after forming a mucoperiosteal flap as per the manufacturer’s protocol. All implant osteotomies, including implant insertion, were executed using the drilling guide. The Conelog implant by Camlog (CONELOG Guide, Camlog, Wimsheim, Deutschland) was inserted. All surgical measures were carried out by a surgeon (SiS) experienced in template-guided technology.

Immediately after implant placement, the obtained primary stability was evaluated by determining the Implant Stability Quotient (ISQ) using the Resonance Frequency Analysis (RFA) (Osstell IDx, Osstell, Göteborg, Sweden). The ISQ is a dimensionless value, which permits conclusions on the micromotion of an implant and consequently on its primary stability and level of osseointegration. The measurement was taken in the mesio-distal and vestibular-oral directions.

The higher this value is, the lower the micromotion. The maximum value to be obtained is 100.

### Registration of the implant position

All implants were preserved with a fixed dental prosthesis. For prosthetic care, the clinical situation was recorded 3 months (±2 weeks) after implant placement using an individual spoon, impression coping, and polyether impression material (Permadyne Garant, 3 M Espe, Seefeld, Germany). One dentist (SiS) made all impressions. After disinfection, a dental technician transferred the impression to a plaster model. The impression coping was supplemented by a screw-on implant analog, and the impression was digitized (3Shape Scanner D 700, 3Shape A/S, Copenhagen, Denmark).

### Superimposing the datasets

The datasets were superimposed using the program Geomagic Studio (Version 9, Geomagic, NC, USA). All data were consecutively analyzed locally and chronologically regardless of their generation by an investigator (LB). The datasets of the digitalized implant impressions were exported as surface files in STL format. The latter represented the clinical implant positions that were achieved. The three-dimensional surface dataset of the implant planning was used as a reference, exported from the treatment planning program (SMOP) in STL format and uploaded to the Geomagic software as well.

Up to a defined structure, the datasets were reduced for the unchanged hard tooth tissue to exclude errors due to changed soft tissue or the deviating implant positions. Based on the RMS value (root mean square, root from the mean of the squares of all deviations), the superimpositions were assessed.

For the planned analyses of the path and angular deviations, the use of auxiliary constructs was necessary, which reflected the 3D structures of the alveolus and the exact position of the implant planning and the clinical implant position achieved. They were produced using the program Surfacer 10.6 (Imageware, Ann Arbor, MI, USA) using simple geometric shapes. The auxiliary constructs were adjusted to the respective implant lengths and diameters and then loaded for mapping in the program Geomagic Studio. In this way, it could be ensured that the axial endpoints and the axial deviation of the implant positions could be determined and standardized for further analysis. This method has been described in detail by Schnutenhaus et al. [17].

The mapped auxiliary constructs, which reflected the key data of the 3D information of the planned and clinical implant position achieved, were loaded for further analysis in the program Surfacer 10.6 Imageware.

### Analysis of the implant position

**Fig. 3:**
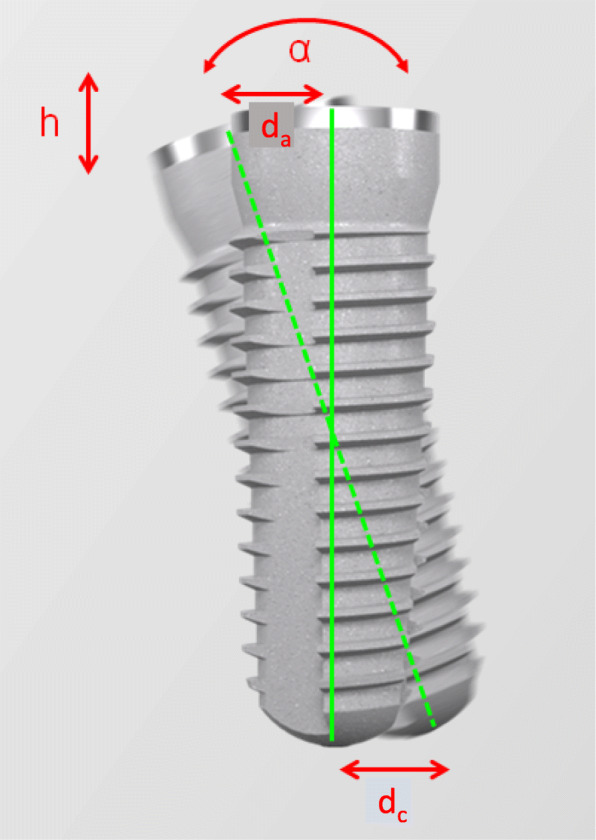
Metric analysis of the deviation between planned and clinical implant position achieved, measured at the center axis. The following was determined: the distance at the implant shoulder (dc), at the implant top (da), the vertical deviation (h), and the angular deviation (ang.)

The metric analysis included the following measurements (Fig. 3):

- Radial deviation: The 3D deviation of the focuses between the implant planning and clinical implant position achieved and measured at the implant shoulder (da) and apex (dc) (corresponds to the Euclidean distance).
- Vertical deviation: The vertical spatial offset, measured at the midpoint of the implant shoulder (h)
- Axial deviation: Angular deviation of the implant axes of the planning and clinical implant position (ang) achieved

The measurement method took place as per the principle of Tahmaseb et al. [18] to facilitate improved comparability with current and future studies.

### Statistical analysis

For continuous variables, the minimum, median, maximum and quartiles were reported. Additionally, the mean and standard deviation were calculated as appropriate. Nominal and ordinal features were described with their absolute and relative frequencies. Group differences were investigated using the Wilcoxon rank-sum test (in the case of two groups) or Kruskal-Wallis test (in the case of three groups). Associations between different continuous variables were investigated via scatterplot and Spearman’s rank correlation. Given the exploratory nature of this study, all statistical outcomes had to be interpreted as hypothesis generating and not as confirmatory. All statistical tests were carried out at the level of α = 0.05 (two-sided). No adjustment was made for multiple testing. The statistical analysis was performed with SAS® Version 9.4.

## Results

### Description of the study population

Of the original 60 patients, 23 were male and 37 were female. Forty-eight patients were included in the study (Table 1).

**Table 1:** Study population

		Total (n)	Group A with augmentation (n/percent)	Group B without augmentation(n/percent)
Total	n	48	25	23
**Sex**	Female	31	17/68.0	14/60.9
	Male	17	8/32.0	9/39.1
**Tooth region**	Anterior tooth	13	6/24.0	7/30.4
	Premolar	28	15/60.0	13/56.5
	Molar	7	4/16.0	3/13.1
**Implant length**	7 mm	1	1/4.0	0/0.0
	9 mm	8	3/12.0	5/21.7
	11 mm	20	13/52.0	7/30.4
	13 mm	19	8/32.0	11/47.8
**Implant diameter**	3.3 mm	1	0/0.0	1/4.3
	3.8 mm	32	16/55.0	16/69.6
	4.3 mm	15	9/45.0	6/26.1

Inserted implants presented according to gender, tooth region, implant length, and implant diameter

The average age was 52 years (24–77 years). Of the 48 evaluated patients, 17 were male and 31 were female. In Group A, the percentage of men was 32%, and the percentage of females was 68%. In Group B, the patients evaluated comprised 39.1% male patients and 60.9% female patients.

### Drop-outs

In two cases, the datasets could not be attributed due to the change in hard tooth tissue by means of the crown preparation before taking the impression. Four implants were modified after removal of the guide on the basis of the deficientprimary stability in its position; two additional data sets could not be located. In the case of two of the original 60 patients, the treatment/study was discontinued due to patients, and two patients became ill and had to be excluded from the study due to their no longer being able to adhere to the schedule. This resulted in a drop-out of 12 cases in total.

### Test specimens

Forty-eight implants were implanted as per the study protocol and could be successfully uncovered and treated after a three-month healing period.

All 48 implants were inserted in the upper jaw in the immediate vicinity of an adjacent tooth or implant where dental support of the drilling guide was always guaranteed.

The distribution to the regions resulted in 13 cases in the anterior teeth, 28 cases in the premolars, and 7 cases in the molar area. All implants were inserted in length and diameter as designed.

### Metric analysis

The mean deviations of all 48 implants were:

- 0.7 mm (0.2–1.7; SD 0.3) at the implant shoulder,
- 1.4 mm (0.3–3.5; SD 0.6) at the implant apex,
- 0.3 mm (0.0–1.4; SD 0.3) the height offset,
- 4.1° (0.4–11.0; SD 2.1) between the axes.

The mean values, minima, maxima and interquartile range (IQR) of the analyzed parameters are listed in Table 2.

**Table 2:** Mean deviations of all 48 implants

		Tooth Region			Alveolar Ridge Preservation		Implant Diameter		Implant Length		
		Anterior Tooth	Premolar	Molar	Yes (Group A)	No (Group B)	3.8 mm	4.3 mm	9 mm	11 mm	13 mm
**Coronal distance**	MV	0.67	0.66	0.73	0.65	0.71	0.65	0.73	0.64	0.62	0.75
	Min	0.38	0.19	0.24	0.19	0.25	0.19	0.24	0.24	0.19	0.50
	Max	1.25	1.66	1.41	1.41	1.66	1.25	1.66	1.66	1.41	1.25
	Median	0.65	0.62	0.62	0.63	0.63	0.62	0.64	0.43	0.59	0.66
	IQR	0.50–0.75	0.49–0.80	0.25–0.99	0.50–0.78	0.46–0.88	0,51–0.79	0.40–0.99	0.32–0.89	0.40–0.77	0.60–0.88
	*P*-value	0.94			0.71		0.64		0.11		
**Apical distance**	MV	1.41	1.31	1.61	1.41	1.35	1,30	1.54	1.06	1.26	1.64
	Min	0.47	0.26	0.48	0.26	0.47	0.26	0.48	0.47	0.26	1.14
	Max	3.07	2.43	3.50	3.50	3.07	3.07	3.50	1.87	3.50	3.07
	Median	1.40	1.28	1.51	1.35	1.30	1.31	1.51	0.94	1.16	1.39
	IQR	1.14–1.58	0.99–1.56	0.90–1.76	1.02–1.52	0.99–1.68	1.01–1.48	0.90–1.87	0.64–1. 48	0.74–1.50	1.25–2.07
	*P*-value	0.57			0.88		0.25		0.02		
**Height**	MV	0.24	0.33	0.18	0.24	0.33	0.28	0.31	0.31	0.25	0.32
	Min	0.05	0.00	0.00	0.00	0.02	0.01	0.00	0.00	0.01	0.05
	Max	0.79	1.4	0.59	0.72	1.40	0.79	1.40	1.40	0.68	0.79
	Median	0.22	0.27	0.06	0.19	0.27	0.24	0.19	0.07	0.24	0.27
	IQR	0.14–0.27	0.09–0.43	0.02–0.42	0.05–0.39	0.12–0.41	0.13–0.37	0.05–0.49	0.03–0.43	0.12–0.35	0.12–0.45
	*P*-value	0.28			0.21		0.65		0.30		
**Angle**	MV	3.8	3.8	5.6	4.5	3,7	3.6	5.2	3.4	3.8	4.5
	Min	0.8	0.4	1.6	0.4	0.8	0.4	1.6	0.8	0.4	2.6
	Max	9.1	8.1	11.0	11.0	9.1	9.1	11.0	5.9	11.0	9.1
	Median	3.8	3.5	5.6	4.2	3.6	3.5	4.9	3.3	3.6	4.2
	IQR	2.6–4.3	2.7–4.5	4.4–5.9	3.1–5.9	2.6–4.9	2.6–4.2	2.7–6.9	2.1–4.8	2.0–4.4	3.2–5.6
	*P*-value	0.14			0.19		0.04		0.29		

Mean values (MV), minima (min), maxima (max), median as well as the interquartile range (IQR), and *P*-values (significance level *p* ≤ 0.05) of the coronal distance, apical distance, vertical deviation, and angular deviation in [mm] according to the “tooth region, alveolar ridge preservation, implant diameter, and implant length” factors

Primary stability was measured in the mesio-distal and vestibular-oral directions. The mean vestibular ISQ value for the ARP group was 63.00 (35–73; SD 8.75) and 64.12 (41–74; SD 7.88) for the control group. The mean mesial ISQ value for the ARP group was 63.48 (35–76; SD 9.60) and 65.15 (43–75; SD 8.30) for the control group.

### Application of statistical tests

The “coronal distance, apical distance, height, and angle” parameters were investigated with regard to the “tooth region, application of hard tissue management measures in the form of ARP, implant diameter, implant length, and primary stability (ISQ)” factors.

The data do not indicate a correlation between the tooth region or measures of hard tissue management in the form of ARP and transfer accuracy.

When the implant length increased, greater coronal and apical deviations were descriptively measured (cf. Table 2). After application of the Kruskal-Wallis test, a significant influence of the implant length on the apical distance could be confirmed (*p* = 0.02). The implant diameter significantly influenced the degree of angular deviation (*p* = 0.04), favoring narrower implants (Ø 3.8 mm).

No significant difference in the mean ISQ values was found between the ARP- and control groups in the vestibular-oral (*p* = 0.460) and mesio-distal (*p* = 0.225) directions. However, the measured values were taken into consideration when applying Spearman’s rank correlation coefficient, where a significant influence of the ISQ on the vertical deviation (*p* = 0.02) became clear. Implants showing a high ISQ value after insertion produced significantly greater vertical deviations compared to the planned implant height. Therefore, bone quality in terms of primary stability is not influenced by the applied ARP measure, but it has an influence on accuracy.

## Discussion

### Methodological critique

The precision of transferring an implant plan to the clinical implant position achieved depends on the extent of the total error. Inaccuracies arise in the course of the individual work steps from the planning phase to implant placement, and the errors add up [19]. It is therefore particularly important to know the maximum deviation for the system used in each case.

Starting with 3D imaging using CBCT, the introduced error depends on the spatial resolution of the imaging data. Distortions or incorrect information in the CBCT image – as, for example, occur due to motion or metal artifacts – negatively influence the image quality and can result in additional inaccuracies in a subsequent superimposition with the associated model scan [20–23]. The most comparable studies for accuracy analysis of guided implant placement use a second, postoperative, CBCT imaging procedure to evaluate the transfer accuracy [24]. The classification takes place either using anatomical reference points or using geometric reference markers installed in an X-ray guide. In such cases, reliable and reproducible seating of the X-ray guide is required for a meaningful outcome in addition to image quality.

The approach selected in this study, which calls for the clinical implant position achieved to be recorded by means of digitalization of the implant impression, has the advantage of a greater degree of achievable precision – in addition to reduced radiation exposure – compared to a renewed, postoperative CBCT imaging. Additional inaccuracies arise while taking impressions and the subsequent digitalization of the situation model and implant impression as well as their overlap with the CBCT dataset respective to the initial model. For digitalization, a lab scanner (3Shape Scanner D 700, 3Shape A/S, Copenhagen, Denmark) was used. This remains unaffected in comparison to the intraoral scanners of toothless parts and increasing distances between scan bodies [25]. With the aid of the treatment planning software SMOP, the datasets obtained were virtually superimposed. Any necessary fine adjustments were made by the dentist. The segmentation of the CBCT data and the experience of the dentist have a significant influence on the accuracy of superimposition [23]. When mapping the datasets of the planning model and clinical implant position (implant impression), the mean RMS error was 31.6 μm (SD 10.2). The mapping errors were thus within the impression accuracy [26, 27].

Intraoperatively, there are a series of additional, potential error sources. Attention should be paid in particular here to the stable and reproducible seating of the drilling guide in the mouth of the patients [6, 28–34].

The tolerance level between the drill sleeve and the drill can result in further inaccuracies [29–31] and influence the precision while transferring [35]. This minimal difference in the diameter inherent in the system is necessary, however, to guarantee that the drill is smoothly guided into the sleeve during implant placement. Camlog indicates an angular deviation of 1.53° for the system used in the study. The mean deviation of four degrees achieved is within the mechanically achievable precision of approximately five degrees and the intrinsic error required for the design and production of the drilling guide [36–38]. .To enable measurements of the situation of implant planning and the clinical implant position achieved relative to each other, it was necessary to map the surface datasets before constructing auxiliary geometries. The advantage of using such standardized auxiliary constructs is the reproducibility. In comparison to other studies with which a manual determination of coronal or apical measurement points was made [39, 40] or no further indication was made for the selected approach [2, 4, 34, 36], a subjective error size can be deemed negligible.

### Comparison of the outcomes to the current literature

Current studies on transfer accuracy present a high degree of inhomogeneity. The difficult comparability to the outcomes of the current literature has already been highlighted in detail in a previous study by Schnutenhaus et al. [41]. In general, in vitro studies result in greater accuracy than cadaver or clinical studies [42]. Furthermore, fully guided implant placement achieves better accuracy of results than partially guided implant surgery [42].

In 2018, Thamaseb et al. published a review paper in which a total of 20 clinical studies were considered. The mean deviation was 1.2 mm (CI 95%: 1.04–1.44) at the implant shoulder and 1.4 mm (CI 95%: 1.28–1.58) at the implant top. The angular deviation was 3.5° (CI 95%: 3.0–3.96). The difference in height at the implant shoulder was 0.2 mm (CI 95%: − 0.25–0.57). More significantly accurate outcomes were produced when evaluating cases with partially edentulous jaws in comparison to edentulous jaws [8]. However, the authors indicated that the validity of the review was limited based on the high level of homogeneity of the study design. Many different surgical techniques were used in the studies included. An explanation for the different outcomes could be the seating of the guide. In comparison to guides supported by the mucous membrane, guides supported by teeth produced significantly more accurate outcomes [34, 43]. In addition, the occurrence of micromotions and the resulting inaccuracies were described for guides purely supported by the mucous membrane even if pins were used for fastening [29–31, 33].

The influence of the operator experience on the accuracy was avoided since only an experienced surgeon performed the implantation. The use of static computer-assisted surgery seems to be only slightly dependent on the experience of the surgeon [44]. A learning curve that leads to better results could not be shown in a clinical study [45] However, the positioning of the template has a significant impact on accuracy. The experience of the practitioner has a significant influence on this factor that influences accuracy [46].

Comparing the outcomes of our study to the outcomes of the review paper by Thamaseb et al., it was found that the deviation was significantly better at the implant shoulder (0.67 mm), and the deviation at the implant apex (1.38 mm) was in the average range. Only the angular deviation turned out to be somewhat worse (at 4.1° to 3.5°).

Schnutenhaus et al., as part of a retrospective study, investigated the conformity of the planned and clinical implant position achieved using the same study design [41]. Factors influencing the outcomes based on a different study design are largely eliminated here. If the outcomes of both studies are compared, it should be noted that they can indeed be placed in a very similar range, which indicates a higher degree of precision for the existing values here. Of particular note are the improved values with the deviation at the implant shoulder and the vertical deviation (on average, 0.5 mm lower deviation in each case). An explanation for this difference could be the existing adjacent natural teeth, which were determined as inclusion criteria in the case of this study. An adjacent natural tooth has a significant influence on the transfer accuracy in the area of the implant shoulder and the height [41]. An adjacent tooth also has a positive, if not significantly positive, influence on the other parameters [41].

### Tooth region

The tooth region did not have any significant influence on the transfer accuracy.

This outcome coincides with the outcomes of Naziri et al., D’haese and Verhamme et al. [1, 33, 47]. No significant correlation between the region, upper and lower jaw, anterior or posterior region, and accuracy achieved were found herein either. Jee-Ho Lee et al., however, achieved significantly higher deviations in the area of the anterior teeth in comparison to the premolar and molar regions [35]. They concluded that a precise working method and an ongoing review of guide seating is of high importance, particularly in the highly esthetic anterior tooth area [35]. In the respective studies, different guide designs were used, and partially edentulous and edentulous patients were treated with implants. As previously mentioned, implant placement in partially edentulous patients provides more accurate outcomes [8]. A direct comparison can therefore be critically classified.

### Surgical measures

Inadequate measures of the ARP generally result in a lower vestibular bone lamella, and as an outcome, the implants are inserted deeper, and more local augmentations are necessary. Measures of the ARP had no influence on the accuracy of the outcomes. Moreover, none of the investigated parameters indicated significant differences between the outcomes of untreated extraction alveoli receiving ARP using a collagen cone.

### Implant diameter and length

Implants were differentiated with diameters of 3.8 mm and 4.3 mm. Implants with smaller diameters indicate a significantly lower angular deviationdeviation. All other parameters were insignificant. There was no further study that addressed the transfer accuracy focusing on the influence of the implant diameter, which interfered with the classification of these outcomes.

The values of this study demonstrated a significant influence of the implant length on the deviation at the implant top (*p* = 0.02). The values for the coronal deviation and for the angular deviation also demonstrated – even if nonsignificantly – a tendency toward a negative influence of an increasing implant length on the transfer accuracy. These values correspond to the outcomes of the current literature. Jee-Ho Lee et al. obtained significantly higher values in the deviation for all determined parameters with the exception of the deviation at the implant shoulder [35]. The deviation at the implant top was also significantly higher for longer implants in the study of D’haese et al. [33]. In a further study, 236 implants inserted by various manufacturers were divided into four groups according to their length: Group 1: 8–9 mm (*n* = 20), Group 2: 10–11 mm (*n* = 112), Group 3: 12–13 mm (*n* = 99), and Group 4: 14 mm (*n* = 5). The outcomes demonstrated that the mesio-distal direction of the implants from Group 1 at the implant shoulder (*p* = 0.006) and at the apex (*p* = 0.013) were significantly more precise than in Groups 2 and 3 [1].

### Implant stability quotient (ISQ)

The postoperative measurement of the ISQ using resonance frequency analysis provides information on the primary stability attained [48] and is indirectly related to the existing bone quality [49]. The evaluation of the measured ISQ values indicates a significant influence of a high ISQ value (> 65) on the vertical deviation of the clinical implant position achieved for the plan (*p* = 0.02) after applying Spearman’s rank correlation coefficient. A possible correlation could exist between a high level of bone quality and the previously achieved high torque and the high primary stability associated with it when inserting the implant. A solid bone could interfere with the complete insertion of the implant without exceeding the recommended torque (Nm). For the additionally investigated parameters, no significant correlations could be determined between a determined ISQ and the transfer accuracy achieved. Instead, no increased deviations were measured with low ISQ values (< 50). This can be interpreted as indicating that the transfer using drilling guides is also reliable in the case of lower bone quality or density. Unfortunately, there is currently no literature that addresses the correlation between ISQ and transfer accuracy.

## Conclusion

The outcomes of this study demonstrate a satisfying accuracy with different insertion regions and different implant lengths/diameters. Longer implants and those with larger diameters generally exhibit worse performance. The hard tissue management measures in the form of ARP have no influence on the accuracy of the implant position. A high torque and an associated, high primary stability can have an effect on the transfer accuracy. Part of the stated hypotheses could therefore be confirmed. ARP or the tooth region has no influence on the accuracy, whereas the implant length and diameter as well as the primary stability have a significant influence on some parameters. Guided implant placement was considered superior to free-hand implant placement. By using drilling guides, the actual surgical procedure can be shortened, and in some cases, augmentation procedures can be avoided. However, the clinical added value has predictable prosthetic outcomes in particular. However, it must be noted that the average values are not achieved in the study; rather, the maximum values are a key factor for the reliability of a system.

## Data Availability

The complete documentation of all patients enrolled in this study belongs to the authors and is available only upon reasonable request.

## Abbreviations

ARP: Alveolar ridge preservation
CAD/CAM: Computer-aided design/Computer-aided manufacturing
CBCT: Cone-beam computed tomography
ISQ: Implant stability quotient
RFA: Resonance frequency analysis
RMS: Root mean square
STL: Standard tessellation language
